# Deformation of Filamentous *Escherichia coli* Cells in a Microfluidic Device: A New Technique to Study Cell Mechanics

**DOI:** 10.1371/journal.pone.0083775

**Published:** 2014-01-02

**Authors:** Yaron Caspi

**Affiliations:** FAS Center for Systems Biology, Harvard University, Cambridge, Massachusetts, United States of America; Dalhousie University, Canada

## Abstract

The mechanical properties of bacterial cells are determined by their stress-bearing elements. The size of typical bacterial cells, and the fact that different time and length scales govern their behavior, necessitate special experimental techniques in order to probe their mechanical properties under various spatiotemporal conditions. Here, we present such an experimental technique to study cell mechanics using hydrodynamic forces in a microfluidic device. We demonstrate the application of this technique by calculating the flexural rigidity of non-growing *Escherichia coli* cells. In addition, we compare the deformation of filamentous cells under growing and non-growing conditions during the deformation process. We show that, at low forces, the force needed to deform growing cells to the same extent as non-growing cells is approximately two times smaller. Following previous works, we interpret these results as the outcome of the difference between the elastic response of non-growing cells and the plastic-elastic response of growing cells. Finally, we observe some heterogeneity in the response of individual cells to the applied force. We suggest that this results from the individuality of different bacterial cells.

## Introduction

Bacterial cells use their peptidoglycan cell wall [1], [2] and cytoskeleton [3]–[6] as stress-bearing elements to counter-balance the expansion force of the turgor pressure [5], [7]. For many years it was hard to quantitatively measure the mechanical properties of these elements. A decade ago, atomic force microscopy (AFM) was first used in order to measure the Young's modulus of extracted, rehydrated sacculi of Gram-negative bacteria [8]. Subsequently, AFM has become a standard method to probe the mechanical response of bacterial cells (for reviews see references [9]–[11]). Recent examples include measurements of cell softening after attack by phages [12] and measurements of the viscoelastic response of the cell envelope [13], [14].

One important question that was less considered is the influence of growth processes on bacterial cell mechanics. Why should there be a relation between the two? To answer this question it is important to notice that bacterial morphogenesis is intimately connected to growth [15]–[17]. For example, the curved conformation of *Caulobacter crescentus* is maintained by an asymmetrical insertion of new cell wall material into the two sides of the cell during the growth process [18], [19]. Similarly, the coordinated relaxation of peptidoglycan cross-linking during the growth of *Helicobacter pylori* is responsible for its helical shape [20]. Theoretical analysis suggest that local control of the rate, processivity, and extent of peptidoglycan insertion can be a general mechanism to create a curved conformation [21], [22]. On the other hand, mechanical forces *per se* can also shape bacteria and control their morphogenesis [23], [24]. For example, the elastic properties of the cell wall, and the mechanical forces acting on it from the flagella, completely determine the curved shape of *Borrelia burgdorferi* [25]. Thus, since both growth processes and direct mechanical forces are important for morphogenesis, it is interesting to ask whether there is a direct relation between cell growth and cell mechanics.

Traditional AFM, however, suffers from two limitations that hamper its ability to probe such a relation. First, the immobilization of the cells on a surface may bias the measurements in various ways [26], [27]. Second, the time scale of the measurements is usually slow compared to the bacterial growth rate. Recent advances in high speed AFM can circumvent this time scale limitation [28]. Indeed, in the last few years high speed AFM was used in order to probe in real time the dynamics of an antimicrobial peptide attack on *Escherichia coli* (*E. coli*) [29], as well as the dynamic movement of surface ultrastructures in *Magnetospirillum magneticum* [30]. Still, a direct measurement of cell mechanics parameters in real-time remains challenging. Thus, complementary experimental techniques may be needed in order to probe the relation between cell growth and cell mechanics.

A few years ago, optical tweezers were used in order to study bacterial cell mechanics [31]. In this method, a bead was attached to the cell tip and was pulled using the optical tweezers, while the other end of the cell was immobilized on a glass cover-slide. The results of this measurement suggested that the MreB cytoskeleton contributes at least 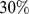 of the flexural rigidity of Gram-negative bacteria. However, once again, the growth of the cells was not taken into account.

A completly different approach was taken by Tuson et al. who recently measured the longitudinal Young's modulus of growing bacterial cells using hydrogels with tunable elasticity [32]. In order to measure the longitudinal Young's modulus, the bacteria were encapsulated in the gel and the deformation of the gel during the growth process was quantified. Their results showed that the longitudinal Young's modulus of different bacteria species is similar, but that unlike the flexural rigidity, the longitudinal modulus is unaffected by MreB depolymerization. These findings highlight the importance of the growth process in the parameter space of the cell mechanics. However, due to the nature of this method it can not be applied in order to probe the lateral stiffness of growing bacteria.

**Figure 1:**
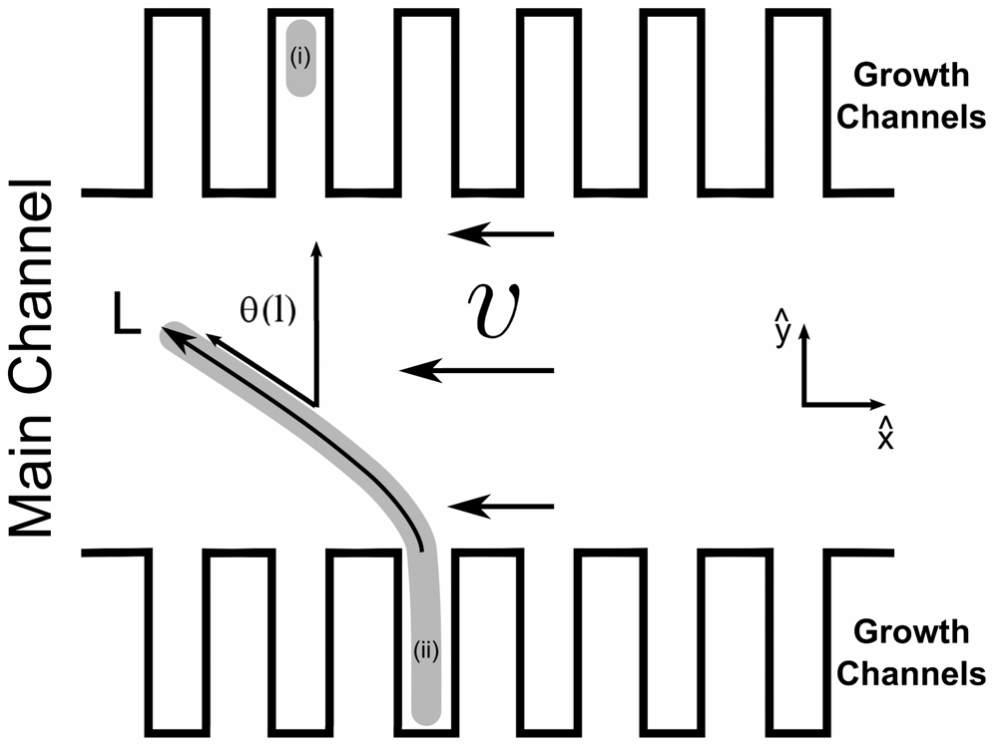
Sketch of the experimental setup. Cells, e.g. cell (i) and (ii), grew in a microfluidic device consisting of dead-end growth channels that were connected at their open end to a large main channel. If cell division is blocked, the cells grow as filaments that penetrate into the main channel. A fluid flow with velocity 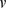 and a profile similar to the one depicted created a hydrodynamic force that deformed the cells, as is shown for cell (ii). The magnitude of the force was controlled by the infusion rate. For analysis, the arc length (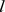) was measured from the point of connection between the growth channels and the main channel (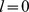) to the tip of the cell (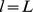). To characterize the shape of the cell, the angle profile (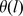) was calculated (see Materials and Methods).

Here we present a simple and readily accessible approach using a microfluidic device to probe bacterial cell mechanics in real time and to study its relation to growth on the single cell level. In the last decade, microfluidic technology has become increasingly important for the biological toolbox [33]. Numerous studies have used it in order to study Eukaryotic cell mechanics (for review see [34]). We utilized its advantages in order to develop a setup to study the relations between growth and cell mechanics of rod shaped bacterial cells. Our device consists of a set of dead-end channels (indicated as ''growth channels'') that are connected at their open end to a large flow channel (''the main channel'' - see figure 1 and figure S1). We grow *E. coli* cells into filamentous form inside our microfluidic device. The growth channels serve as supporting points that circumvent the need for a permanent immobilization step. Fluid flow in the main channel enables us to apply hydrodynamic forces on the cells in order to deform them and simultaneously to create different environmental conditions to control their growth conditions. Thus, we can directly probe the relation between the growth of the filamentous cells and their deformation. We used this approach in order to measure the flexural rigidity of the cells, and to show that at low forces, a smaller force is needed in order to deform growing cells to the same extent as non-growing ones due to the plasticity of the growing cells.

## Results

### Lateral deformation of non-growing cells

**Figure 2:**
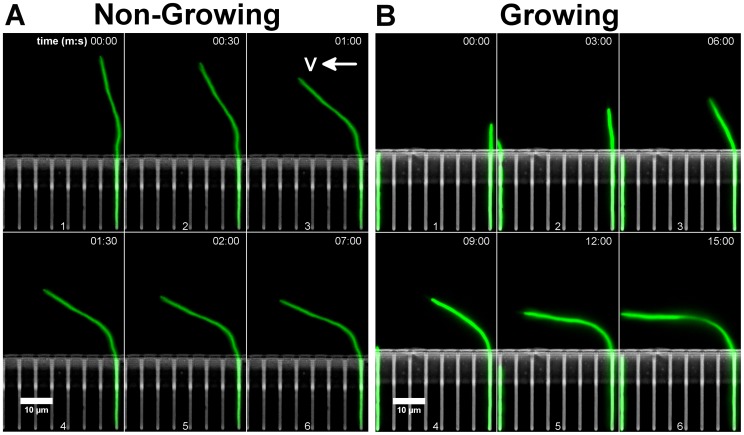
Deformation of a non-growing cell (A) and a growing cell (B). Flow was from right to left. Infusion rate was 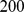 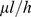. For the non-growing cell (A), the cell did not deform along its entire length outside of the growth channel. Rather, the deformation seems to occur over a restricted area around the exit from the growth channel. Note the time difference between panel (6) and panels (1-5), showing that after 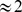 minutes, the cell obtained its steady state conformation. For the growing cell (B), as the cell grew into the main channel, and when a sufficient force was applied, it deformed. Initially, the deformation resembled the bending of the non-growing-cells. As the cell grew longer, it deformed more from a straight conformation. Finally, it grew horizontally.

**(i) Non-growing** ***E. coli*** **cells deform elastically.** In order to grow cells into a filamentous form inside the microfluidic device we loaded them from an LB culture and induced the expression of SulA under the Plac promoter. SulA belongs to the SOS response system of *E. coli, and* inhibits the formation of the FtsZ ring and thus of cell division [35]–[37]. During filamentation, fresh LB media was constantly infused into the device until the cells were 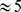 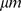 shorter than the growth channels. At that point, the infusion of the media was stopped, and the cells were left to grow as straight rods penetrating into the main channel. When the part of the filamentous cells in the main channel was 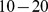 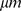 long, we briefly infused buffer A into the device (pure M9 salts, sodium azide, IPTG - see materials and methods). The double effect of the carbon depletion and of the blocking of ATP synthesis stopped cell growth almost immediately. We maintained IPTG in buffer A to assure that even during the time it takes to stop the growth, cell division will continue to be inhibited. It should be noted that buffer exchange resulted in hydrodynamic forces that could modify the conformations of the cells. Thus, we developed a protocol that minimized the flow during the exchange, yet promised that the cells will stop growing over a short period of a few minutes (see materials and methods). Still, even using this protocol some cells did deform before their growth stopped. We further studied only cells with almost straight conformation after growth arrest and a size of the part in the main channel of less than 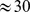 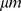. After the cells stopped growing, as was inferred from comparison of time lapse images, they were allowed to relax for an additional 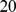 minutes in buffer A but without a flow, to ensure that no residual growth still continued. Finally, the mechanical response of the cells was studied by infusing buffer A at a certain flow rate and recording the cells' deformation. An example of the deformation of a cell under a lateral force is shown in figure 2(A). After the application of the force, the cell quickly deformed until it reached a steady state conformation (note the large time difference between panels (5) and (6)).

**Figure 3:**
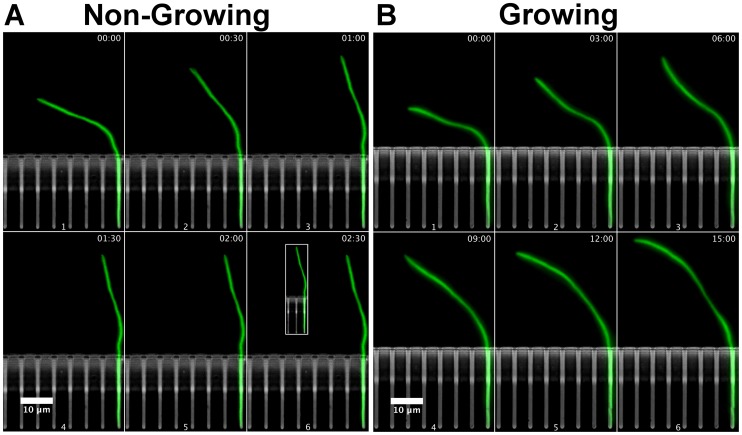
Relaxation of growing and non-growing cells. (A) Relaxation of a non-growing cell to its native conformation after the flow was minimized. The cell relaxed to its initial conformation after 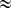
1.5 min. Inset - the conformation of the cell before the force was applied. (B) Changes in the deformation of a growing cell after the flow was stopped. The cell straightened up a bit but continued to grow in a curved conformation. In both cases, the original flow was 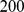 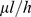 and it was stopped or minimized after the first time point as described in the text.

The fact that non-growing cells deformed under force led us to ask whether they will also retain their predeformed conformation when the force is removed. If they do not, the deformation is probably due to an irreversible change in the cell structure; whereas if they do, the deformation is probably an elastic one. In order to discriminate between these two possibilities, we relaxed the force by reducing the flow rate to a minimum value of 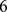 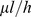 and allowed the fluid to simultaneously exit from one of the inlets as well as from the outlet. We used this procedure rather than just stopping the flow completely, in order to avoid differences in the baseline force due to transient flows in the device. A typical example of the relaxation of a single cell is shown in figure 3(A). As can be seen, when the force was removed, the cell recovers its predeformed conformation within a short period (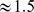 min, inset to figure 3(A)). We believe that this time scale represents mainly the relaxation time of the flow in our device rather than any internal time scale that is related to the cell (re)deformation.

**Figure 4:**
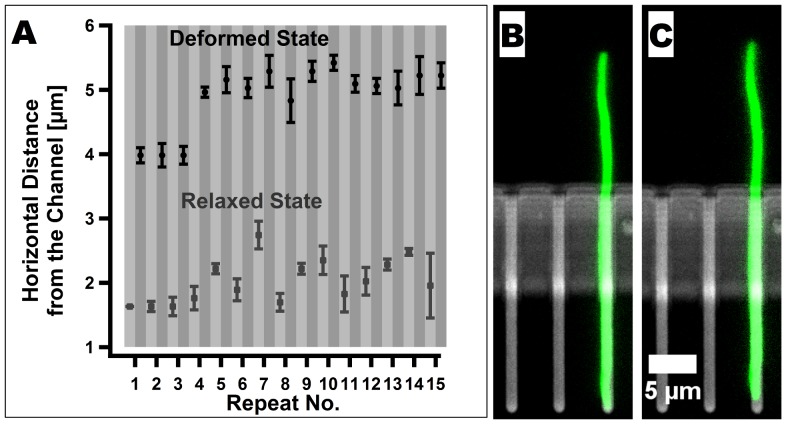
Reversibility of the deformation of non-growing cells. (A) Horizontal distance from the tip of a cell to the growth channel for a repeated application of flow of 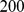 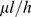 followed by a recovery phase. Altogether, a force was applied on the cell 15 times. After each time, the flow was reduced to 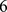 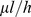 and the fluid was allowed to flow into the second inlet as well as to the outlet. The small difference in the horizontal distance after three recoveries phases results from a small sliding of the cell during the application of the force. (B) - (C) A non-growing cell conformation before (B) and after (C) 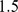 hours of experiencing flow at a rate of 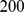 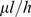, followed by a recovery phase similar to the one that is described above.

We further demonstrated that non-growing cells relax to their native conformation by performing two supporting experiments. First, we studied the deformation of a cell using a recurring application of force followed by a recovery phase. A plot of the horizontal deviation of the tip of the cell from the position of the center of the growth channel is shown in figure 4(A). Second, we applied a flow of 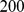 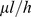 on a non-growing cell for an hour and a half and then allowed it to recover (see figure 4(B)-(C) and figure S2). In both cases, the cells relaxed to their predeformed state after the force was removed. Thus, we conclude that non-growing cells deformed elastically in our device and did not experience plastic deformation under the range of forces that were used.

**Figure 5:**
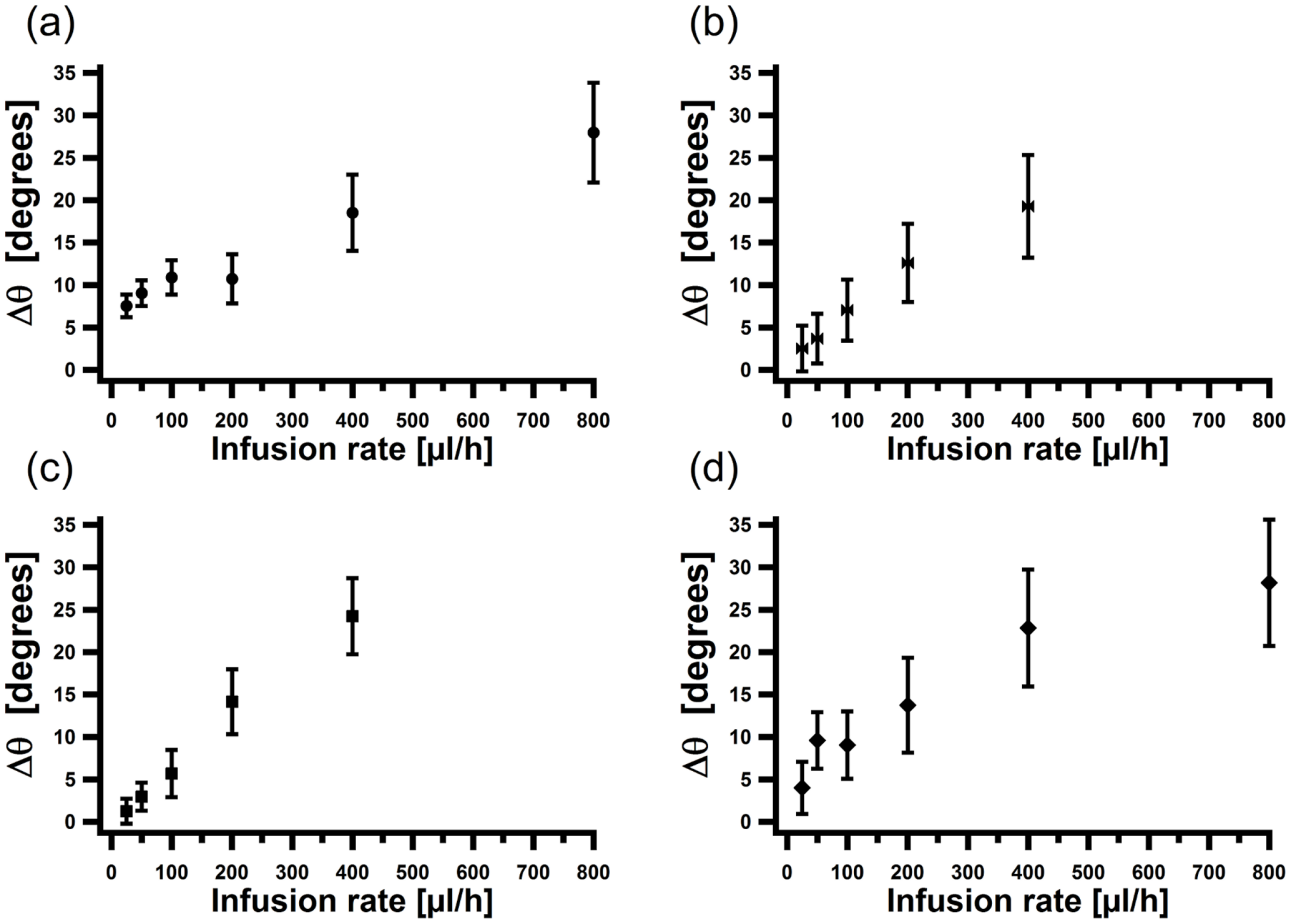
Analysis of the deformation of non-growing cells. The difference of the angle 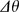 between the tip of the cell (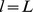) and its basal end (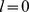) for 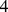 cells with arclength in the main channel of (a) 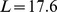 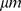 (b) 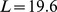 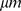 (c) 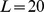 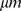 and (d) 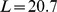 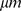 as a function of the infusion rate.

**(ii) Characterization of the deformation of non-growing cells.** Having shown that non-growing cells deform elastically in our device we turned to study their deformation in more details. First, we looked at the deformation of the non-growing cells under repeated re-occurrence of deformation and relaxation processes, similar to the one described above, while increasing the infusion rate in the device (and therefore the force) from a value of 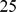 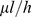 to 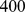 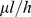 (or 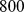 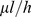 if the cell did not escape). Using a custom Matlab code and an ImageJ macro (see materials and methods) we extracted the shape of the midline of the cells and characterized each cell by its angle profile 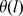 between the tangent to a midline segments and the 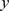 axis (see materials and methods, figure 1 and figure S3). For reasons that are explained below we analyzed the extent of the deformation using the value of 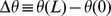, where in both cases the value is defined by a fit of the tangent vector for the first or last few microns of the cell to a straight line. A collection of the results for 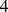 different cells is shown in figure 5. As can be seen, 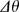 increased linearly as the infusion rate (and hence the force) is increased. A linear fit to the results of these four cells plus one other (data not shown) gave a value of 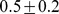 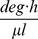 for the incline of 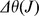, where 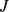 is the infusion rate. This result is compared below with a similar result for the deformation of growing cells.

**Figure 6:**
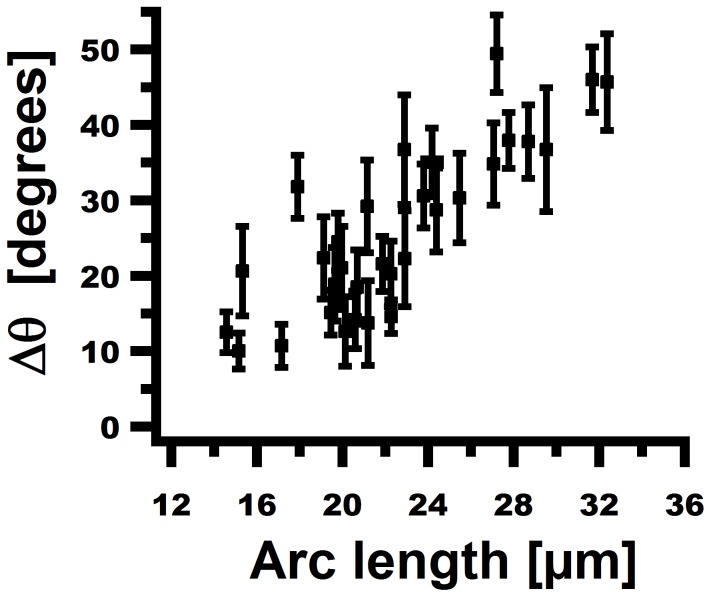
Analysis of the deformation of non-growing cells. The difference of the angle 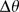 between the tip of the cell (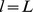) and its basal end (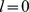) for 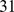 cells with arc length in the main channel of 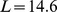 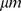 to 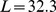 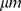 as a function of the arclength under a flow of 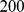 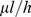.

Next, we studied the deformation of 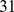 cells with arclength of 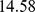 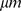 to 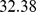 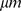. Results of the relation between 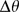 and the arclength (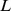) are shown in figure 6 (see also table S1). As can be seen, 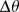 increased linearly as a function of 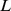. Notably, we observed some cell-to-cell variation in the propensity to deform under force (about 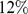 as inferred from standard deviation of a linear fit to the result). Hence, under the same flow value, several cells deform more than others. In principle, this can result from: (i) influence of the initial growing phase of the cells before they were injected into the microfluidic device, (ii) deviations in the flow field between different cases, or (iii) variability of the lateral stiffness of individual cells. In order to check the first possibility we plotted 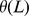 as a function of the O.D. of the cells upon injection into the device but did not find any correlation between the two parameters (data not shown). We checked the second possibility by infusing fluorescent beads (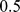 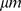) while cells populated the device and following the influence of the cells on the beads trajectories. We observed deviations from straight trajectories, and hence laminar flow, only adjacent to the boundaries of the cells (See movie S1 movie S2). Thus, the presence of cells did not influence the flow profile in the device to a significant extent. We therefore favor the last explanation that cell-to-cell variability is responsible for the spread in the results. However, since we did not measure directly the flow field profile in each case, we cannot rule out flow field deviations as the origin of the cell-to-cell variability.

**(iii) Calculation of the Flexural Rigidity.** Since the cells in our device behave elastically, and since their arclength is much larger than their diameter (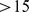), we can calculate their flexural rigidity (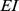), which is the product of the Young's modulus 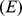 and the second moment of inertia (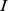), from the classical elastic theory of the bending of thin rods [38]. In practice, we solved the elastic equations for 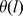 using the Matlab boundary problem solver bvp4c with the flexural rigidity as the free parameter. For the calculation we used the external hydrodynamic force in the explicit form for a flow in a closed duct with a high aspect ratio as given by Gondret et al. [39] (see text S1 and text S2). A note should be given, though, to the choice of boundary conditions. Obviously, the tip of the cell in our devices is free (both its position and its direction are arbitrary). In contrast, the base of the cell follows a supported boundary condition, i.e. it can slide at the point of support but cannot undergo a transverse displacement. The fact that the base of the cell is supported is evident since: (i) at high infusion rate cells tend to escape out of the growth channels by slipping, and (ii) the direction of the tangent vector at the base of different cells had a somewhat different value and was evidently different than zero (see table S1). However, for a supported point, like for a free point the direction of the tangent vector cannot be deduced from predefined conditions. Thus, we solved separately for each cell the elastic equations, and used as a boundary conditions the fitted directions of the tangent vector at the base and the tip of the specific cell as were extracted by the custom written Matlab function (see materials and methods section). In fact, the cell can go through some lateral displacement if the growth channel is a bit larger than its diameter, as is always the case. However, this displacement was neglected since it is much smaller than the lateral displacement for 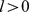. In order to calculate the error bar of each measurement, we repeated the calculation of the flexural rigidity twice; once with the base angle and the tip angle equals to their fitted values plus the error of the fits, and a second time with the base and tip angles equals to their fitted values minus the error of the fits (see figure S4). Altogether we calculated the flexural rigidity of 31 cells when the applied infusion rate was 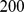 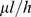. From these calculations we find 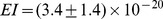 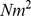, a value that is of the same order of magnitude as the one that was measured by Wang et al. [31]. The error represents standard deviation for the values of different cells (see table S1).

### Deformation of growing cells

**(i) Deformation profile is similar to that observed for non-growing cells.** We next examined the deformation process of growing cells. An example of the deformation of a single cell under a flow of 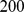 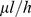 is shown in figure 2(B). As the cell grew out of the growth channel, it experienced an increasing force. Initially, it sustained the force and grew as a straight filament. However, when it reached a length of about 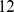 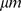 the force became too large to sustain, and it started to deform. As the cell continued to grow, the deformation angle increased. Finally, it grew horizontally in the main channel. Note that the deformation mode of the growing cell looks similar to the one of non-growing cells. Thus, looking only on the state of the cell at one time point (see for example figure 2(b) panel 4) and without previous knowledge of the cell history or growth state, it is very hard to determine in a simple way if this specific cell is a growing one or not. However, in this case one cannot infer the flexural rigidity of the cells by a calculation similar to the one that was done for non-growing cells, since cells synthesize and degrade their sheath during the process of the deformation.

It should be noted that, similar to the case of non-growing cells, growing cells were also observed to have certain cell-to cell variability in the behavior of 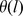 (see figure S5). This behavior further supports our conclusion that there might exist some cell-to-cell variability in the mechanical properties of the cells' sheath.

**(ii) Growing cells do not relax completely after the force is removed.** In order to stress the difference in the deformation process of growing and non growing cells, we examined the relaxation of growing cells when we stopped the infusion. An example of the behavior of a growing cell when the deformation flow is reduced from 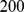 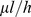 to zero is shown in figure 3(B). Immediately after the flow stopped (panel (2) of the figure) 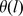 became smaller and the cell became less curved. Yet, instead of relaxing to a straight configuration as a non-growing cell, it continued to grow in a curved way. This behavior shows that the deformation of a growing cell resulted from two complementary mechanisms: an elastic deformation due to the instantaneously acting force, and a permanent plastic deformation of the cell structure as a result of forces that acted on it in the past. It should be noted that this description does not contradict previous suggestions that growth can lead to the generation of straight conformation [19], [21], [22], [40]. In those studies, the mechanisms that were suggested operate on a longer time scale of several doubling time of the bacteria, whereas here we discuss the behavior over a much shorter time scale of 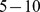 min (the doubling time is 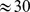 min in our case).

**Figure 7:**
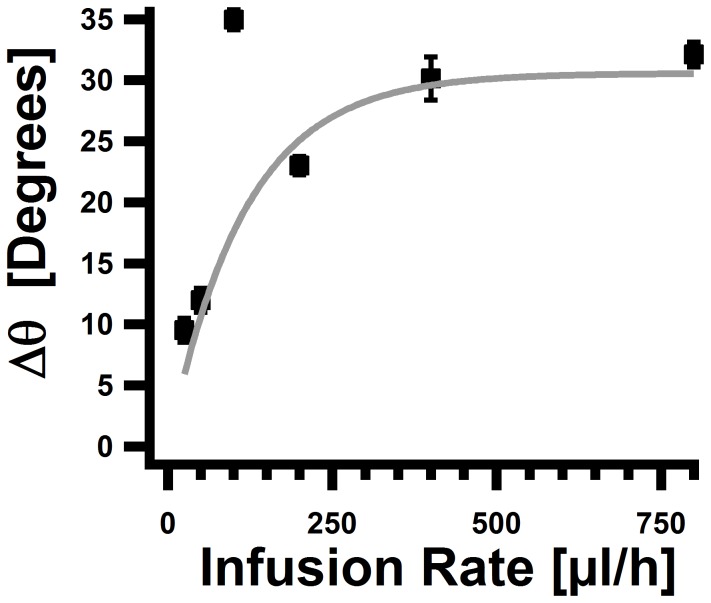
Analysis of the deformation of non-growing cells. The difference of the angle 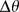 between the tip of the cell (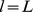) and its basal end (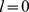) for growing cells. The value of 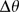 was derived from a global fit to a straight line of 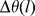 for 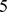, 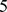, 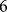, 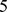, 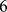 and 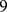 cells at infusion rates of 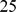, 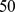, 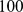, 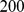, 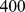 and 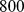 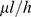, respectively (see figure S5 ). Gray line - fit to an exponential function.

**(iii) Comparison of the deformation of growing and non-growing cells.** In order to compare the propensity of growing and non-growing cells to deform under force we analyzed the deformation of growing cells under various values of the infusion rate (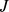). Since the deformation pattern of growing cells can be separated into two phases, one where the cell is straight, and one where it curves (see figure 2 and figure S5), we recorded the behavior of several cells at each infusion rate and fitted globally 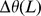 for different values of the infusion rate (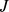) during their growth process to a straight line. From these fits we extracted the value of 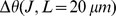. Results are shown in figure 7, where the error bars represent standard deviation of the values derived from the standard deviation of the fits to the straight lines. As can be seen, 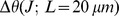 increases linearly at low values of 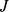 and then saturates as the infusion rate (and hence the force) is increased. We fitted this function to the form 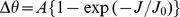 and found a values of 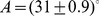 and 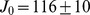 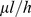.

this function for 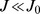 we find 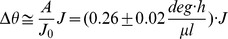. Comparing this value to the one that was found for non-growing cells we conclude that the force that is needed to deform growing cells to the same extent as non-growing cells is approximately two time smaller for low values of the force (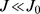).

## Discussion

Adopted from elastic theory and materials science origins, deforming a biological material is now a standard way to study its mechanical properties. For example, the flexural rigidity of microtubules and cells were measured by deflection using optical tweezers [31], [41]. In particular, the force that results from hydrodynamic flow was used previously from both a theoretical and experimental point of view in order to study elastic properties [42], [43]. We presented an experimental technique to study the lateral deformation of filamentous cells using hydrodynamic forces in a microfluidic device, and demonstrated this experimental method by studying the deformation of growing and of non-growing filamentous *E. coli* cells. This approach is somewhat different from previous measurements since unlike common materials, growing cells can deform their sheath. Our study has led to three findings:

(i) We have used our device to calculate the flexural rigidity of non-growing *E. coli* cells and found it to be 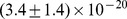 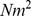. It should be noticed that Wang et al. calculated a value of 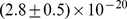 for the flexural rigidity of native *E. coli* cells and a value of 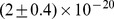 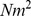 for E. coli cells treated with MreB [44]. Most probably, the slightly larger flexural rigidity value that was calculated by us for the native *E. coli* cells results either from the fact that the non-growing cells were not perfectly straight before the application of the force, or from a slight over estimation of the force that acted on the cells due to their tendency to grow out of focus. Still, our calculation serves as further evidence for the order of magnitude of the elastic strength of Gram-negative bacterial cells.

(ii) We estimate that in our setup, due to the plastic response of growing cells, the force that was needed to deform non-growing cells was approximately two times larger than the one that was needed to deform growing cells to the same extent at low values of the force, i.e. when the deformation angle of the non-growing cells is close to zero. We would like to stress that both growing and non-growing cells minimized their mechanical energy by aligning with the flow field. The difference between them is that a growing cell can remodel its sheath according to the stress field that it experiences during its growth process. Thus, in addition to an elastic response, growing cells possess the ability to deform irreversibly in a plastic manner. Due to this mechanism a cell could reduce the mechanical energy cost that is associated with its deformation. In practice, this resulted in a larger deformation angle of the growing cells relative to the case of non-growing cells under the same force when the force was small. Consistent with this idea, we showed that non-growing cells relax to their initial conformation after the force was removed whereas growing cells did not relax to a straight conformation if a force was constantly impinged on them during their growth. Hence, whereas non-growing cells behaved like a stretched spring that released its mechanical energy when the force was removed, growing cells behaved like a combination of a stretched spring and a natively curved rod that did not have a mechanical energy cost for their curved conformation. These measurements are among the first to estimate this effect quantitatively.

It should be noted that part of the difference between the deformation behavior of growing and non-growing cells may be attributed to the difference in osmolality between the LB buffer that was used during deformation of growing cells (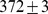 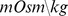), and that of buffer A (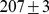 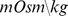), which was used during the deformation of non-growing cells. However, inhibition of growth with either sodium azide or with rifampicin in LB, showed quantitatively the same picture (data not shown). We therefore believe that most of the effect results from the interplay between elasticity and plasticity rather than from the difference in osmolalities. Indeed, we observed a stiffening of cells when pure water was used in order to inhibit growth, but the effect of osmotic pressure on the mechanics of non-growing cells is left for future experiments.

(iii) we have noticed some cell-to-cell variations in the propensity to deform. During the last decade, it has been shown that bacterial cells possess a variety of systems with a large cell-to-cell heterogeneity such as the cell-surface antigens they express or their chemotactic swimming behavior [45]. It was suggested that they utilize this cell-to-cell heterogeneity as a survival strategy. Recently, Fantner et al. have measured the activity of antimicrobial peptide on individual cells and saw a large heterogeneity in the dynamics of induced bacterial cell death [29]. Similarly, Yao et al. saw three different modes of bulge formation for different cells within an isogenic *E. coli* population after treatment of the cells with cephalexin [46]. It is most probable that these heterogeneities were the outcome of differences within the cell walls, the coupling of the cell walls to the outer membrane or the coupling of the cell walls to the cytoskeleton of individual bacterium. Note that the SD over the mean of the flexural rigidity that was measured by Wang et al. (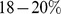) was also rather large [44]. Our results, albeit only preliminary, support such conclusion that individual cells vary with respect to their shell structure and motivate future experiments on the individuality of bacteria with respect to their mechanical strength. Such studies may shed further light on the ability of single cells to adapt to their environment or to resist antibiotic treatments.

Finally, we want to mention that the experimental method that is presented here can be used in the future to understand the mechanics of other bacilli bacteria such as Gram-positive and acid-fast ones (i.e. mycobacteria that posses an especially thick cell wall layer). It can also be used to understand the mechanical effects of drugs or mutations that impair cell wall synthesis or cytoskeleton assembly. In his comprehensive review about bacterial shape, Kevin Young wrote: ''What We Need...we need to be able to manipulate shape apart from other biochemical changes'' [47]. The device and results presented here are one answer to Young's call.

Note inserted during proof: after the submission of this article we learned about two experiments similar to ours. First, Nezhad et al. used the flow in a microfluidic device to measure the Young's modulus of pollen grain [48]. Second, Amir et al. have used essentially the same setup in order to show that E. coli cells deflects either elastically or plastically depending on the duration of the applied force [49].

## Materials and Methods

### Bacterial strain

We used E. coli strain SJ152 derived from a weakly motile MG1655 #6300 strain [50]. The strain was transduced with intC::lambdaPR-YFP to constitutively express YFP [51] and further transformed with plasmid pDB192 [52] containing the SOS protein SulA under the Plac promoter.

### Microfluidic device

We used a previously characterized PDMS microfluidic device [44]. Briefly, the device consists of a two main channels that were connected to two inlets and to one outlet. The cross-section of the main channels was 98 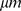 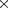
26 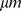. To each channel two sets of 2000 smaller dead-end growth channels were connected from each side. The dimensions of the growth channels were 1.51 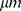 by height, 25 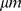 by length and their width varied between 1.2 to 1.6 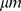. Microfluidic devices were chemically treated before assembly in order to remove uncured PDMS by washing for two hours in Pentane following by two washes for three hours in acetone and drying in fresh air [53]. The device was assembled on a glass bottom Willco dish (Willco Wells BV the Netherlands). To prevent cell adhesion to the PDMS surface, it was passivated before the experiment by incubation for 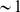 hour at 37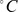 with 2:8:10 ssDNA (Trevigen 9610-5-D) : 10 mg/ml BSA (J.T.Baker A464-02): water.

### Growth Conditions and Microscopy

All experiments were done at 30°C. Cells were grown in test tubes (3 ml in a 14 ml tube) in LB broth (10.0 g Tryptone, 5.0 g Yeast Extract, 10.0 g NaCl per 1 L) + 100 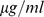 ampicillin (Sigma-Aldrich A9518) to an OD 600 of 0.2–1.1 on a shaker (240 rpm). When the desired OD was reached, the cells were concentrated to an OD 600 of 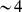, injected into the microfluidic device and the device was incubated until a sufficient number of growth channels were populated. Next, it was washed with prewarmed LB to clear residual bacteria from the main channels.

Microscopy experiments were done using a Nikon eclipse Ti microscope equipped with a constant temperature incubator (In Vivo Scientific) and a Nikon intensilight as an illumination source. Images were collected using a Nikon 100x PlanApo 1.4 NA objective, and a CoolSNAPHQ cooled CCD camera (Photometrics). Images were grabbed into the computer using Nikon NIS-Elements program. Harvard apparatus pumps PHD and pump 33, operated using a customized LabView programs, were used in order to infuse media at various rates as indicated. The device was placed under the microscope and LB broth + 150 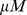 IPTG (Goldbio.com I2481C5) with or without 0.1 mg/ml BSA was used to induce filamentation. Time lapse fluorescence microscopy combined with Z stack acquisition (3 figures separated by 2 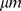 each) was used to record the cells` shape as a function of time. Bright field images were recorded at intervals during the experiment in order to identify the location of the growth channels.

Deformation of non-growing cells was studied in Buffer A that lacks any carbon source and contain: 1x M9 salts (BD Difco 248510), 0.5–1 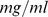 sodium azide (ACROS 26628-22-8) and 150 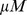 IPTG. Fast buffer exchange with minimal force application during the exchange was achieved by halting the infusion of the LB broth and infusing buffer A for 10 minutes in pulses of 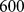 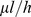 of 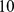 sec, followed by the same procedure with 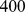 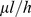 for 5 min, and finally wash with 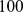 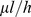 for half an hour. During the buffer exchange phase the direction of the flow in the LB broth inlet was reversed so that it serves as a second outlet.

### Image analysis

Images were opened using the BioFormats plugin of ImageJ (Rasband WS (1997-2012) ImageJ. Bethesda, Maryland, USA,: National Institutes of Health. URL http://imagej.nih.gov/ij/.). Cell boundaries were extracted with the MultiThresholder plugin using the Renyi Entropy or Li methods. A custom code written in Matlab (MATLAB (2011) version 7.13.0 (R2011b). Natick, Massachusetts: The MathWorks Inc.) was used in order to find the mid-line of each cell at each time point by dissecting the cell boundary into two and calculating the average location of the two halves [54]. Following Wiggins et al. [55], points were distributed every 0.5 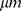 along the midline. For non-growing cell, a custom code written in Matlab was used in order to calculate the flexural rigidity from the profile 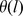 as described in details in Text S2. All graphs were prepared using IgorPro (IgorPro (2009) Version 6.1.2.1. Lake Oswego, OR, USA: WaveMetrics, Inc.).

## Supporting Information

Figure S1 **Outline of the microfluidic device that was used for the measurements.** The device consisted of two inlets and one outlet that were integrated into two parallel main microfluidic channels. At each side of the two main channels, a set of 2000 smaller, dead-end growth channels were connected. Filamentous cells first grew inside the growth channels until they penetrated into the main channels where they experienced hydrodynamic force that resulted from the flow.
(TIF)

Figure S2
**Recovery of a non-growing cell to its native conformation.** Reproduction of Figures 6(B)-(C) form the main text in a format of an overlay of the non-growing cell conformation before (green) and after (red) 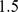 hours of experiencing flow at a rate of 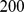 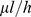 following by a recovery phase.(TIF)

Figure S3
**An example of a non-growing cell deforming under increasing force.** After each case, the infusion in the main channel was minimized and the cell was allowed to recover to its intact conformation. Panel 1 intact conformation; panels 2-6, forces of 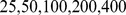 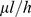, respectively.(TIF)

Figure S4
**An example of the analysis of the deformation of a non-growing cell.** (a) midline position for a non-growing cell before (gray) and after (black) the application of a force (infusion rate is 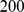 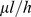). Straight grey line represents the end of the growth channel. (b) Results of the custom code written in Matlab for the analysis of the deformation. Black line - conformation of the part of the cell from (a) in the main channel in reduced coordinates for which the total arclength of the cell is 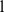. Dark gray line - conformation of a cell as deduced from the elastic equations for which the angle at the base and the angle at the tip are equals to these of the analyzed cell. Light gray lines - same as the dark gray line with the angle at the base and the angle at the tip equals to the fitted values plus and minus the error of the fits respectively.(TIF)

Figure S5
**Profile of the deformation of growing cells.** (a)-(f) 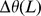 for infusion rates of 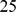, 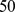, 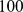, 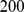,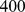 and 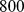 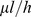 and for 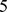, 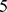,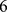, 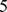,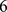 and 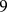 cells, respectively. 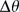 was recorded at different time points during the growth of cells when a force was constantly applied on them. Gray lines are fits to a linear function of the monotonically increasing part of 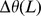.(TIF)

Figure S6
**Velocity profile in the main channel.** (a) Example of the velocity profile in the main channels. The infusion rate was 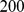 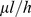. Different colors represent measurements of the velocity profile at the left and right sides of the two main channels. For each position, next to the growth channels a short boundary zone was observed where the velocity decreased. Further away from the growth channels a plateau of the value of the velocity was observed. (b) Velocity over the maximum velocity at that height. Black curve theoretical value. Gray curve average of the four curves from (a).(TIF)

Figure S7 **Flow velocity as a function of the infusion rate.** Measured plateau velocity of beads as a function of the infusion rate. Blue circles and red triangles represents the results of two different experiments. For comparison theoretical values, based on the measured dimensions of the main channels, and assuming a homogeneous flow profile are shown (green dots). The theoretical value is larger than the measured value, a fact that is consistent with the non-homogenous flow profile inside the main channel.
(TIF)

Figure S8
**Theoretical flow profile in the device based on Gondret et al.** [**39**] **.** (A) Predicted normalized flow profile in a close duct with a cross section of 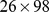 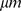. Velocities were normalized to the maximal velocity at the center of the channel. (B) Predicted normalized flow profile in a close duct, with the above mentioned dimensions, in the relative part of the channel that the *E. coli* cell in our experiment may occupy. Velocities were normalized to the maximal velocity at the center of the channel. (C) Predicted flow velocities over the velocity at the same point at a height of 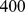 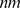 in the relative part of the channel that the *E. coli* cell in our experiment may occupy. Baseline was chosen to be at half of the cells diameter, thus giving an estimation of the velocity that a longitudinal segment of the *E. coli* cells experience relative to the velocity we measured.(png)

Movies S1
**Examples of the flow around cells.** 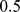 micron beads (red) were infused into the microfluidic device in the presence of cells (green). Note how the trajectories of the beads are influenced by the presence of the cells only adjacent to the cells themselves. Exposure time of each frame in the red channel 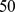 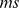. Delay between frames 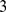 min, infusion rate 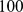 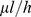.(AVI)

Movies S2
**Examples of the flow around cells.** 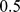 micron beads (red) were infused into the microfluidic device in the presence of cells (green). Note how the trajectories of the beads are influenced by the presence of the cells only adjacent to the cells themselves. Exposure time of each frame in the red channel 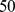 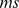. Delay between frames 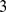 min, infusion rate 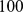 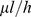.(AVI)

Movies S3
**Trajectories of beads in empty device.** Trajectories of 0.5 micron beads in the microfluidic device as recorded by a TIRF setup at infusion rate of 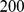 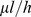 (see text S1). Exposure time of each picture 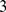 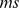.(AVI)

Table S1
**Characterization of the measured cells.** The arclength (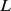), the angle at the exist from the growth channels {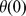}, the angle at the tip of the cell {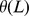} and the calculated flexural rigidity (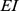) for 31 cells that were deformed using an infusion rate of 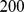 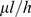.(PDF)

Text S1 **Characterization of the flow field.**
(PDF)

Text S2 **Calculation of the flexural rigidity.**
(PDF)
