# Supplementary figures and images for: Deformation of Filamentous Escherichia coli Cells in a Microfluidic Device: A New Technique to Study Cell Mechanics
Source: PLoS One. 2014 Jan 2;9(1):e83775. doi: 10.1371/journal.pone.0083775 (PMC3879274; doi:10.1371/journal.pone.0083775)

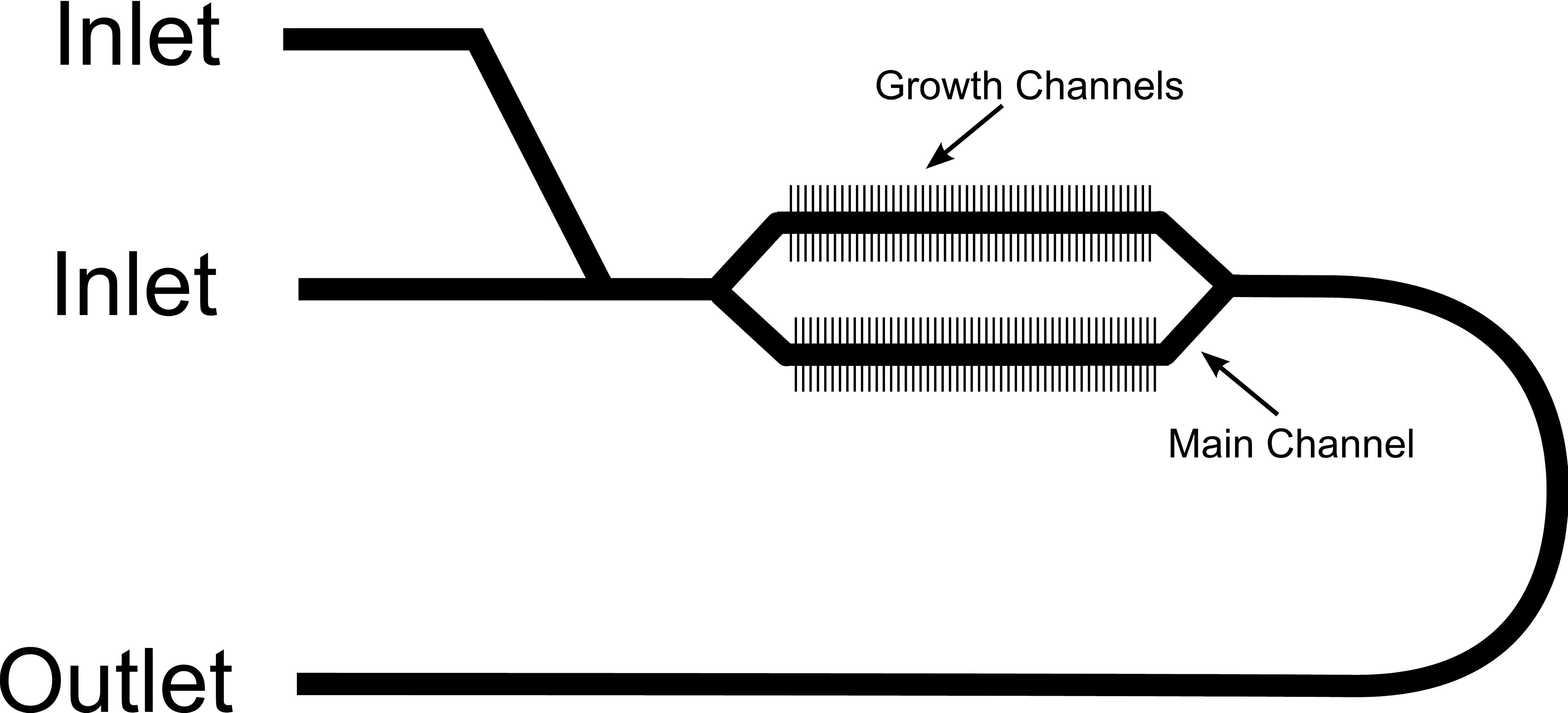

Supplement: Figure S1 — Outline of the microfluidic device that was used for the measurements. The device consisted of two inlets and one outlet that were integrated into two parallel main microfluidic channels. At each side of the two main channels, a set of 2000 smaller, dead-end growth channels were connected. Filamentous cells first grew inside the growth channels until they penetrated into the main channels where they experienced hydrodynamic force that resulted from the flow. (TIF) [file pone.0083775.s001.tif]

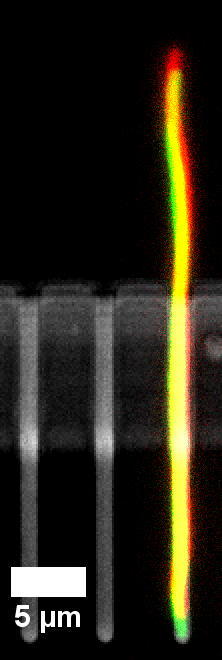

Supplement: Figure S2 — Recovery of a non-growing cell to its native conformation. Reproduction of Figures 6(B)-(C) form the main text in a format of an overlay of the non-growing cell conformation before (green) and after (red) hours of experiencing flow at a rate of following by a recovery phase. (TIF) [file pone.0083775.s002.tif]

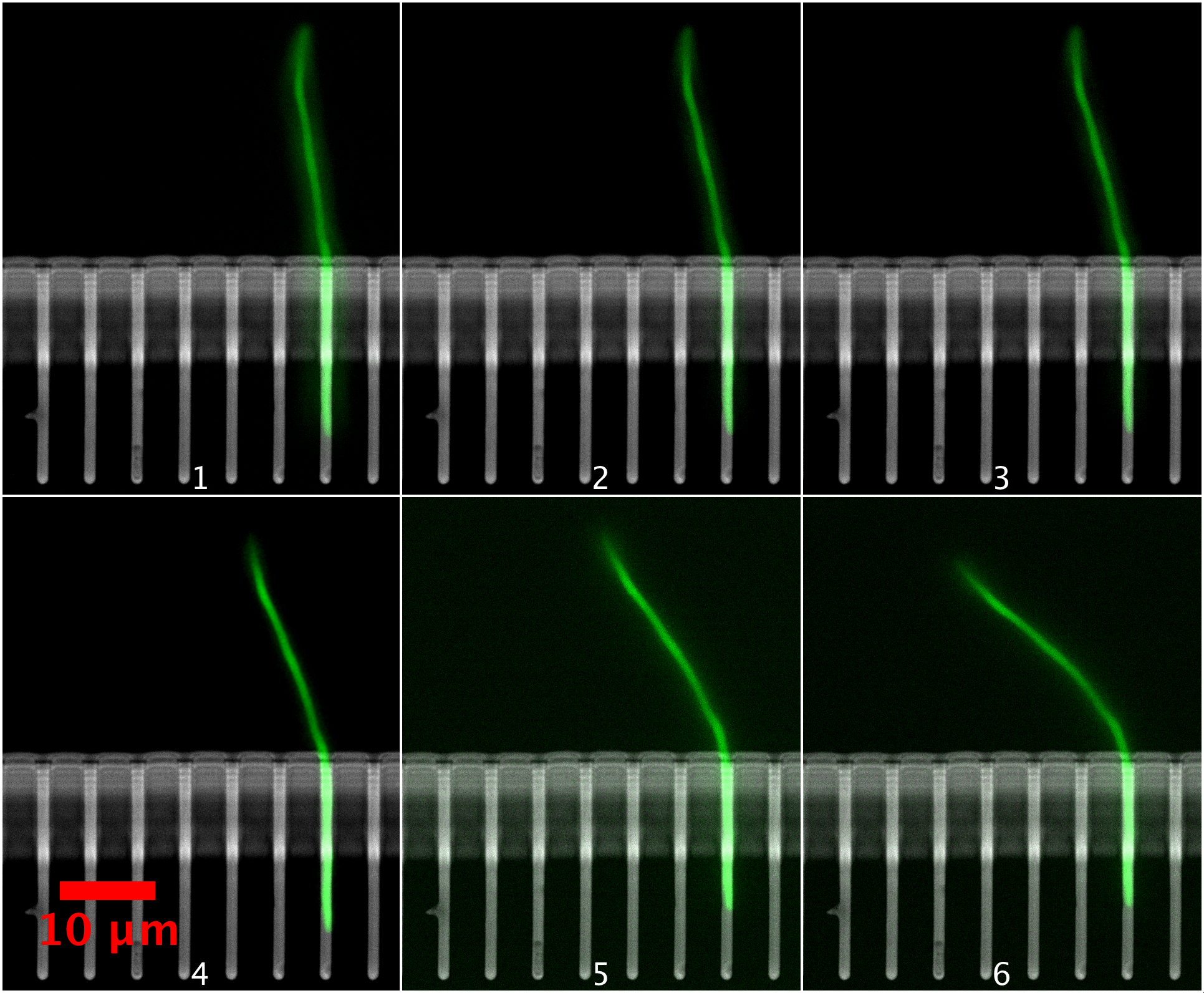

Supplement: Figure S3 — An example of a non-growing cell deforming under increasing force. After each case, the infusion in the main channel was minimized and the cell was allowed to recover to its intact conformation. Panel 1 intact conformation; panels 2-6, forces of , respectively. (TIF) [file pone.0083775.s003.tif]

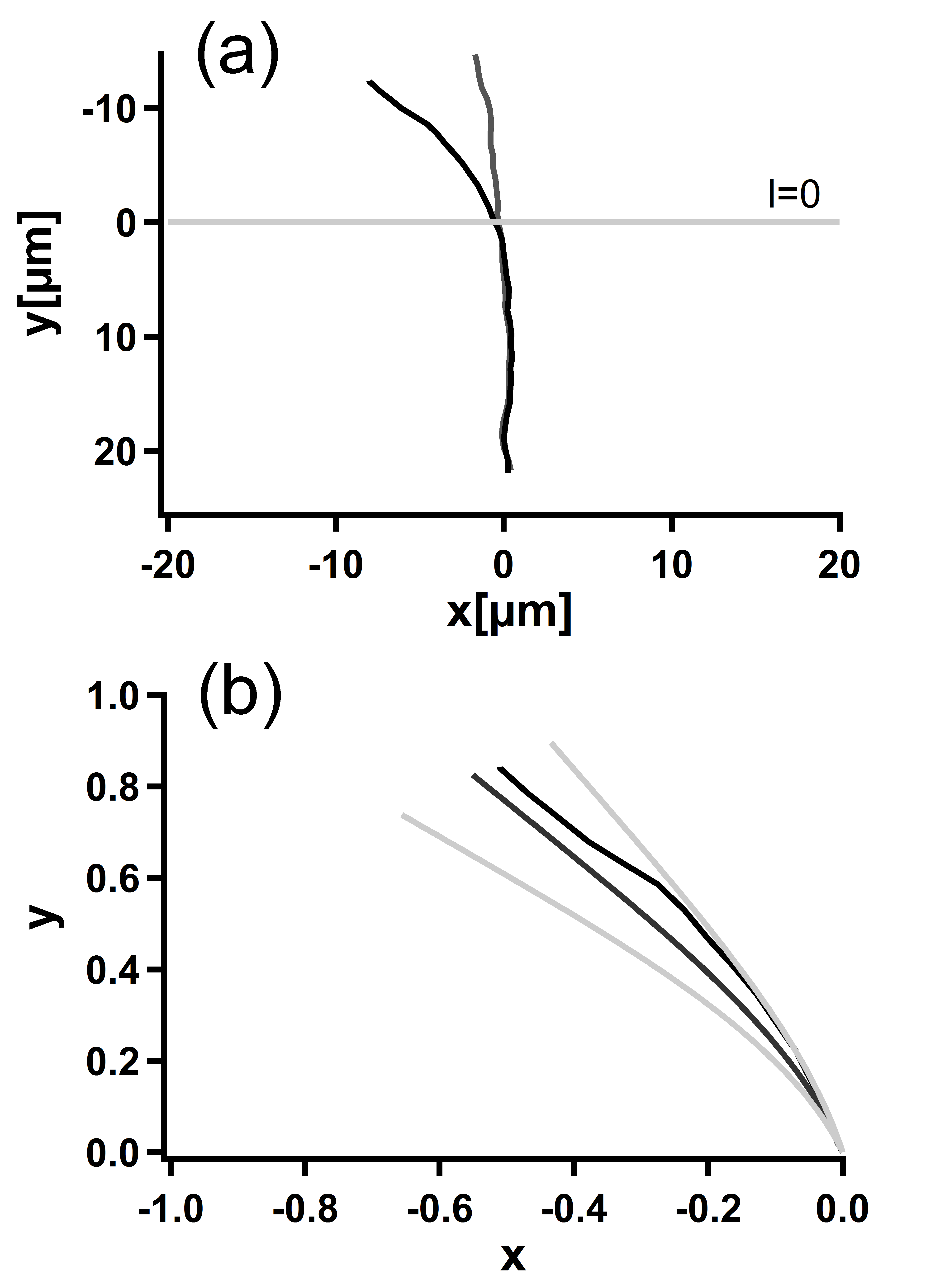

Supplement: Figure S4 — An example of the analysis of the deformation of a non-growing cell. (a) midline position for a non-growing cell before (gray) and after (black) the application of a force (infusion rate is ). Straight grey line represents the end of the growth channel. (b) Results of the custom code written in Matlab for the analysis of the deformation. Black line - conformation of the part of the cell from (a) in the main channel in reduced coordinates for which the total arclength of the cell is . Dark gray line - conformation of a cell as deduced from the elastic equations for which the angle at the base and the angle at the tip are equals to these of the analyzed cell. Light gray lines - same as the dark gray line with the angle at the base and the angle at the tip equals to the fitted values plus and minus the error of the fits respectively. (TIF) [file pone.0083775.s004.tif]

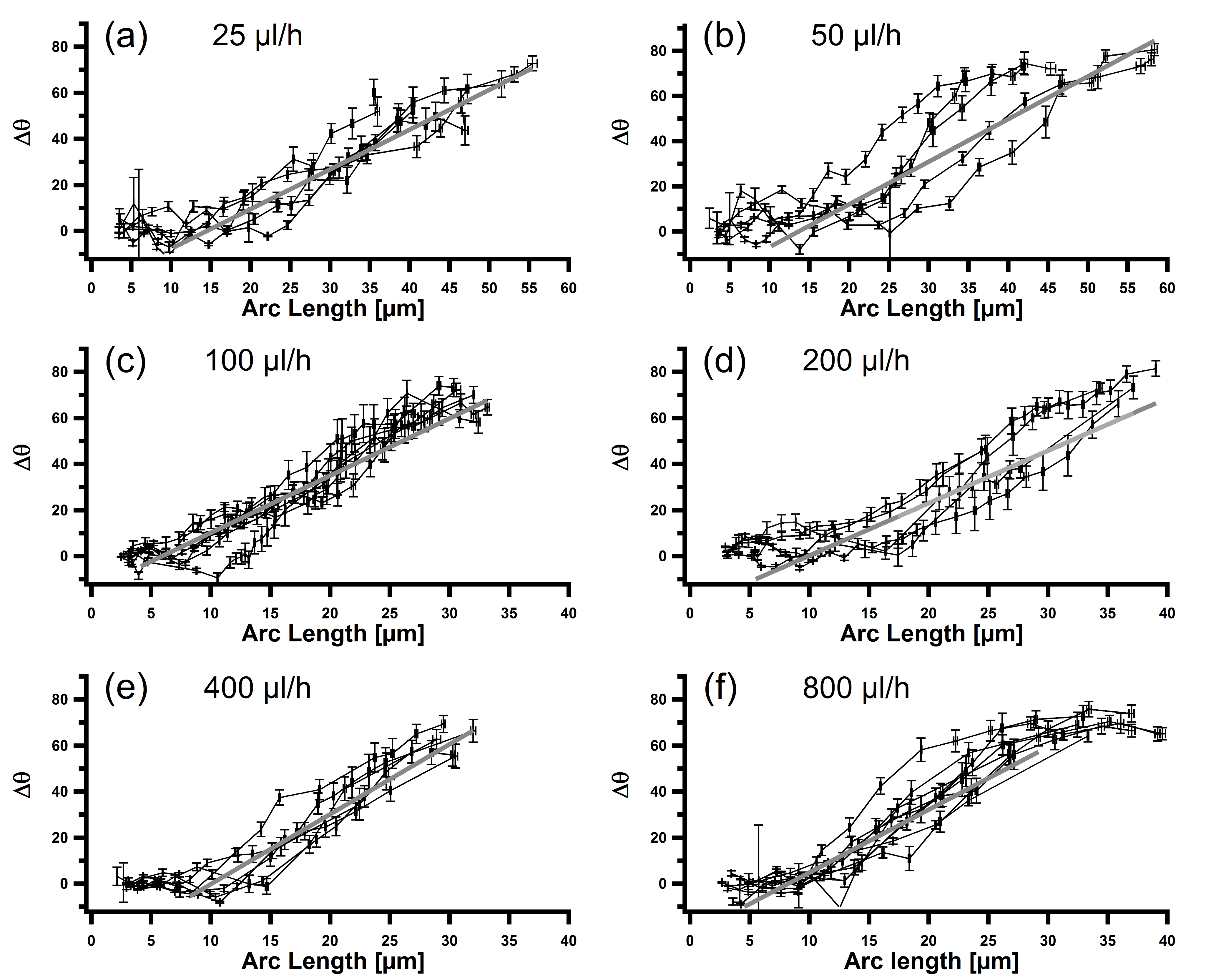

Supplement: Figure S5 — Profile of the deformation of growing cells. (a)-(f) for infusion rates of , , , , and and for , ,, , and cells, respectively. was recorded at different time points during the growth of cells when a force was constantly applied on them. Gray lines are fits to a linear function of the monotonically increasing part of . (TIF) [file pone.0083775.s005.tif]

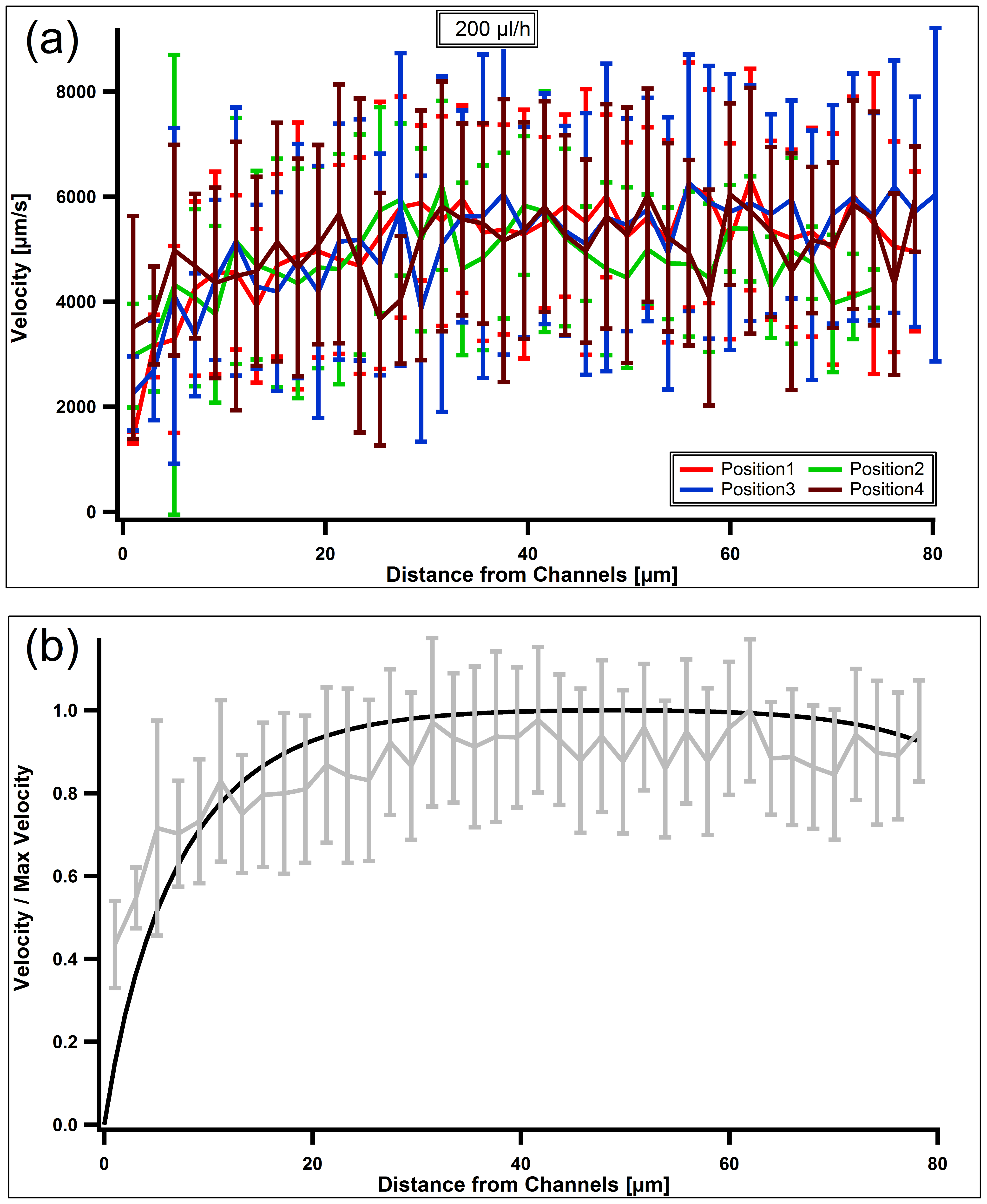

Supplement: Figure S6 — Velocity profile in the main channel. (a) Example of the velocity profile in the main channels. The infusion rate was . Different colors represent measurements of the velocity profile at the left and right sides of the two main channels. For each position, next to the growth channels a short boundary zone was observed where the velocity decreased. Further away from the growth channels a plateau of the value of the velocity was observed. (b) Velocity over the maximum velocity at that height. Black curve theoretical value. Gray curve average of the four curves from (a). (TIF) [file pone.0083775.s006.tif]

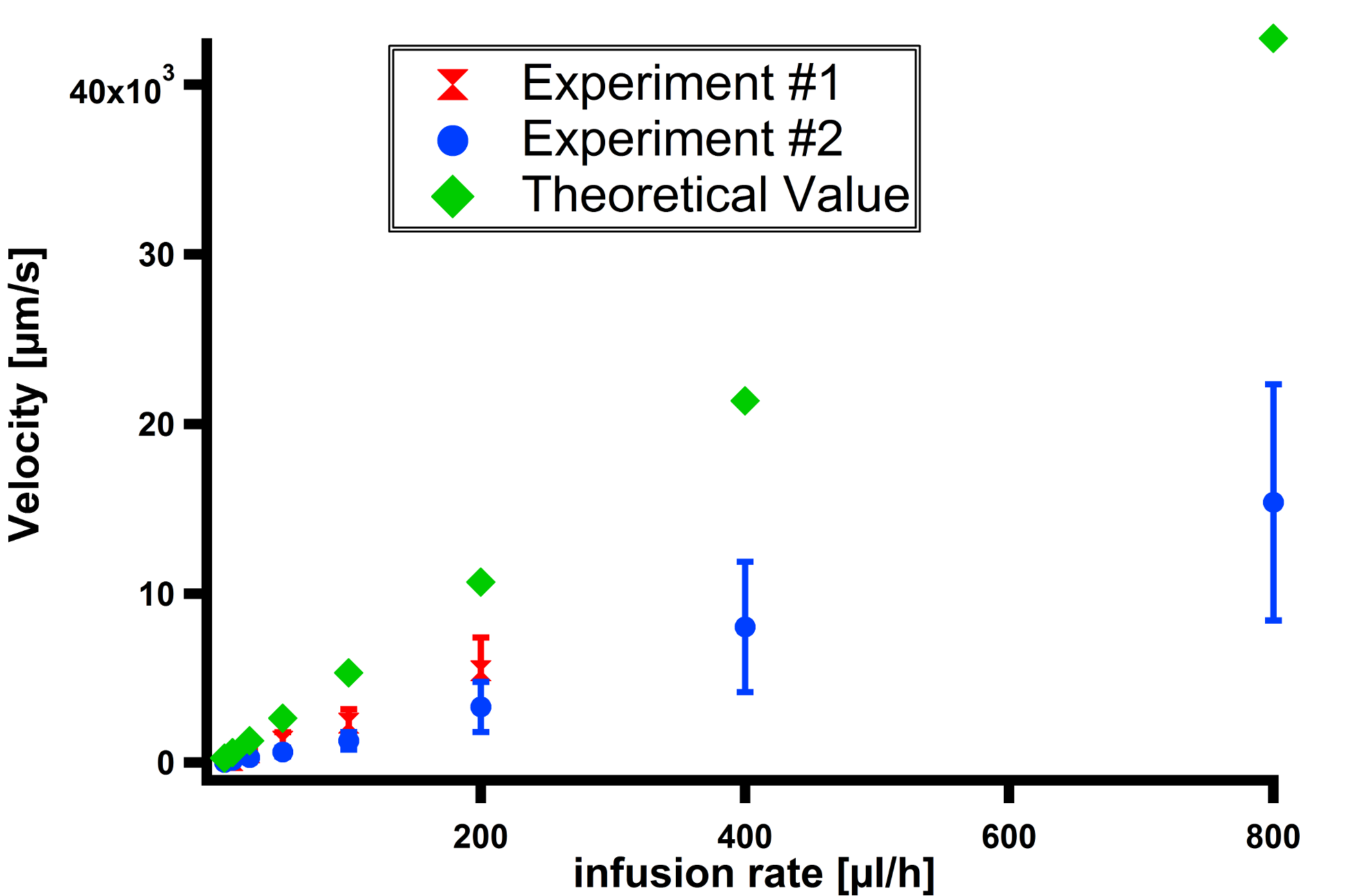

Supplement: Figure S7 — Flow velocity as a function of the infusion rate. Measured plateau velocity of beads as a function of the infusion rate. Blue circles and red triangles represents the results of two different experiments. For comparison theoretical values, based on the measured dimensions of the main channels, and assuming a homogeneous flow profile are shown (green dots). The theoretical value is larger than the measured value, a fact that is consistent with the non-homogenous flow profile inside the main channel. (TIF) [file pone.0083775.s007.tif]
