# Supplementary material for: Deformation of Filamentous Escherichia coli Cells in a Microfluidic Device: A New Technique to Study Cell Mechanics
Source: PLoS One. 2014 Jan 2;9(1):e83775. doi: 10.1371/journal.pone.0083775 (PMC3879274; doi:10.1371/journal.pone.0083775)
Supplement: Table S1 — Characterization of the measured cells. The arclength (), the angle at the exist from the growth channels {}, the angle at the tip of the cell {} and the calculated flexural rigidity () for 31 cells that were deformed using an infusion rate of . (PDF) [file pone.0083775.s012.pdf]

# Deformation of Filamentous *Escherichia coli* Cells in a Microfluidic Device: a New Technique to Study Cell Mechanics - Supporting Table S1

| Cell # | $L$ ($\mu m$) | $\theta(0)$ (degrees) | $\theta(L)$ (degrees) | $EI$ ($\times 10^{-20}$ $Nm^2$) |
|---|---|---|---|---|
| 1 | $14.6 \pm 0.09$ | $7.4 \pm 0.5$ | $19.9 \pm 2.6$ | $2.4 \pm 0.98$ |
| 2 | $15.1 \pm 0.08$ | $11.4 \pm 1$ | $21.4 \pm 2.2$ | $3.5 \pm 1.1$ |
| 3 | $15.3 \pm 0.09$ | $18.4 \pm 0.9$ | $39.5 \pm 4.8$ | $1.4 \pm 0.74$ |
| 4 | $17.2 \pm 0.14$ | $14.2 \pm 1.1$ | $25.0 \pm 2.7$ | $4.6 \pm 1.86$ |
| 5 | $17.9 \pm 0.11$ | $24.6 \pm 1.4$ | $56.4 \pm 3.9$ | $1.0 \pm 0.41$ |
| 6 | $19.1 \pm 0.17$ | $22.3 \pm 2.0$ | $44.7 \pm 5.1$ | $2.3 \pm 1.17$ |
| 7 | $19.6 \pm 0.14$ | $27.7 \pm 1.9$ | $46.5 \pm 4.5$ | $2.8 \pm 1.43$ |
| 8 | $19.4 \pm 0.13$ | $18.9 \pm 1.6$ | $34.0 \pm 2.5$ | $4.3 \pm 0.85$ |
| 9 | $19.8 \pm 0.15$ | $16.7 \pm 1.6$ | $41.2 \pm 3.5$ | $2.5 \pm 0.69$ |
| 10 | $20.0 \pm 0.18$ | $23.3 \pm 1.9$ | $44.4 \pm 5.2$ | $2.8 \pm 1.55$ |
| 11 | $20.1 \pm 0.12$ | $19.7 \pm 1.7$ | $32.3 \pm 4.3$ | $5.8 \pm 3.13$ |
| 12 | $20.6 \pm 0.14$ | $23.5 \pm 1.2$ | $37.7 \pm 3.7$ | $5.1 \pm 2.44$ |
| 13 | $20.7 \pm 0.16$ | $30.0 \pm 2.0$ | $48.5 \pm 4.5$ | $3.3 \pm 1.66$ |
| 14 | $21.2 \pm 0.13$ | $25.7 \pm 1.7$ | $39.4 \pm 5.3$ | $5.7 \pm 4.2$ |
| 15 | $21.2 \pm 0.23$ | $27.4 \pm 4.4$ | $56.6 \pm 4.3$ | $1.9 \pm 0.53$ |
| 16 | $21.9 \pm 0.15$ | $22.8 \pm 2.1$ | $44.4 \pm 3.0$ | $3.6 \pm 0.84$ |
| 17 | $22.3 \pm 0.17$ | $21.6 \pm 1.6$ | $41.8 \pm 4.0$ | $4.3 \pm 1.65$ |
| 18 | $22.9 \pm 0.21$ | $25.6 \pm 2.2$ | $62.3 \pm 6.9$ | $1.6 \pm 1.52$ |
| 19 | $22.3 \pm 0.17$ | $13.8 \pm 1.1$ | $28.3 \pm 1.9$ | $7.2 \pm 1.29$ |
| 20 | $22.9 \pm 0.19$ | $28.8 \pm 2.3$ | $52.5 \pm 4.7$ | $3.3 \pm 1.63$ |
| 21 | $23.8 \pm 0.22$ | $23.6 \pm 2.8$ | $54.2 \pm 3.1$ | $2.8 \pm 0.57$ |
| 22 | $24.4 \pm 0.18$ | $23.5 \pm 1.4$ | $52.2 \pm 5.4$ | $3.3 \pm 1.91$ |
| 23 | $24.9 \pm 0.19$ | $23.4 \pm 1.7$ | $61.3 \pm 4.0$ | $2.1 \pm 0.86$ |
| 24 | $25.5 \pm 0.15$ | $14.2 \pm 0.9$ | $44.5 \pm 5.9$ | $4.2 \pm 2.36$ |
| 25 | $27.1 \pm 0.33$ | $26.0 \pm 2.5$ | $60.8 \pm 4.9$ | $2.9 \pm 1.57$ |
| 26 | $27.2 \pm 0.21$ | $19.8 \pm 1.3$ | $69.2 \pm 5.0$ | $1.7 \pm 1.19$ |
| 27 | $27.8 \pm 0.24$ | $15.1 \pm 1.5$ | $53.1 \pm 3.4$ | $3.8 \pm 0.99$ |
| 28 | $28.7 \pm 0.26$ | $23.9 \pm 2.1$ | $61.7 \pm 4.4$ | $3.3 \pm 1.5$ |
| 29 | $29.5 \pm 0.23$ | $17.8 \pm 1.8$ | $54.6 \pm 8.0$ | $4.5 \pm 3.92$ |
| 30 | $31.7 \pm 0.33$ | $20.7 \pm 1.6$ | $66.7 \pm 4.0$ | $3.2 \pm 1.44$ |
| 31 | $32.4 \pm 0.24$ | $17.9 \pm 0.9$ | $63.6 \pm 6.3$ | $3.8 \pm 3.0$ |

Table S1:
