# Supplementary material for: Deformation of Filamentous Escherichia coli Cells in a Microfluidic Device: A New Technique to Study Cell Mechanics
Source: PLoS One. 2014 Jan 2;9(1):e83775. doi: 10.1371/journal.pone.0083775 (PMC3879274; doi:10.1371/journal.pone.0083775)
Supplement: Text S1 — Characterization of the flow field. (PDF) [file pone.0083775.s013.pdf]

Deformation of Filamentous *Escherichia coli* Cells in a Microfluidic Device: a New Technique to Study Cell Mechanics - Supporting Text S1

Characterization of the flow field

We used TIRF microscopy in order to characterize the flow profile in the main channels next to the coverslip. Images were recorded using a Zeiss Inverted TIRF microscope at the Harvard Center for Biological Imaging (Zeiss TIRF 3). Fluorescence beads (Invitrogen FluoSpheres F8888 0.5 μm) were diluted 1:2000 into MilliQ water and were infused into the device using the Harvard Apparatus pump 33 at different infusion rates. Images were recorded using a photometric Evolve camera, a Lasos 77 argon laser and a 100x 1.46 NA αplan apochromat objective. Zeiss Axiovision program was used to collect data. Exposure times and laser power were varied as a function of the infusion rate in order to obtain tracks of the fluorescence beads that will fill most of the camera field of view. Bead tracks were analyzed using a custom code written in Matlab.

The velocities of the beads (v) for infusion rates (I) of $6 - 800$ $\mu l/h$ were extracted from the recorded length of the track of the beads, the magnification and the exposure time (see movie S3). Altogether we preformed two technical replica and in each replica the velocity profile was measured at four locations at each side of the two main channels. The results show that the average velocity increased as a function of the distance from the growth channels and reached a plateau (See figure S6(a)); As expected, the value of the velocity at the plateau increased linearly as a function of the infusion rate (See figure S7). Note that such profile fits the theoretical prediction of a duct with a large aspect ratio [1] as is draw in figure S6(b).

In order to obtain a better characterization of the actual flow profile in the device, we used a known formula for a laminar flow profile in a close duct with the aspect ratio of our microfluidic device [1]. The results of a simulation of the profile is shown in figure S8(A). Obviously, different parts of the cell experienced different flow rates and hence different forces. To go behind this qualitative description we note that the cells in our experiments tend to bend upward during their growth. The tip of the cells may reach to a height of about $5 - 6$ μm above the main channel bottom (we have used Z stack in order to obtain the cells' projected contour). For each cell the effect was different, and some cells bent upwards more than others. In most cases the tip height (which was the highest part of the cell) was less than 4 μm. In figure S8(B) we plot the relative flow in the part of the main channel that was occupied by our cells in the case where a cell is the longest that we used (i.e. ≈ 30 μm) and it bends upwards the most (i.e. ≈ 4 μm). As can be seen, the flow profile that cells experienced along their longitudinal dimension is highly inhomogeneous.

In order to better estimate the range of flow rates that the cells actually experienced, we draw in figure S8(C) the expected flow rate divided by the flow at half of the height of a cell that sits flat on a surface (400 μm). The fact that our cells tend to grow upwards, cause them to feel a flow at their tip that is

up to $3-4$ times larger than the flow on a tip of a cell that sits flat on the surface. We have used this information in order to calculate the flexural rigidity of non-growing cells as is described in the supporting text S2.

References

[1] Gondret, P., N. Rakotomalala, M. Rabaud, D. Salin, P. Watzky. 1997. Viscous parallel flows in finite aspect ratio Hele-Shaw cell: Analytical and numerical results. Phys. Fluids 9:1841–1843
