# Supplementary material for: Deformation of Filamentous Escherichia coli Cells in a Microfluidic Device: A New Technique to Study Cell Mechanics
Source: PLoS One. 2014 Jan 2;9(1):e83775. doi: 10.1371/journal.pone.0083775 (PMC3879274; doi:10.1371/journal.pone.0083775)
Supplement: Text S2 — Calculation of the flexural rigidity. (PDF) [file pone.0083775.s014.pdf]

Deformation of Filamentous *Escherichia coli* Cells in a Microfluidic Device: a New Technique to Study Cell Mechanics - Supporting Text S2

Calculation of the flexural rigidity

The elastic equations

From the theory of elasticity it is known that the two equations governing the bending of an elastic rod are [1]:

$$\frac{\partial \overrightarrow{F}_{elastic}}{\partial l} = -\overrightarrow{K} \tag{1}$$

and

$$\frac{\partial \overrightarrow{M}}{\partial l} = \overrightarrow{F}_{elastic} \times \hat{t} \tag{2}$$

where $\overrightarrow{F}_{elastic}$ is the elastic force, $\overrightarrow{K}$ is the external force per unit length, $\overrightarrow{M}$ is the moment of the internal stresses and $\hat{t}$ is the tangential unit vector to the rod.

In our case, the external force is the hydrodynamic force that is applied by the fluid flow. In the case of a very low Reynolds number the hydrodynamic force on an object in the context of resistive force theory can be written as [2]:

$$\overrightarrow{F}_{hydro} = \boldsymbol{\xi}\overrightarrow{v} = (\xi_\perp \hat{n}\hat{n}^T + \xi_\| \hat{t}\hat{t}^T)\overrightarrow{v} \tag{3}$$

where $\overrightarrow{v}$ is the velocity field and $\boldsymbol{\xi}$ is the drag tensor, $\hat{t}$, in the context of thin rods, is the tangent unit vector to the rod center line, $\hat{n}$ is the perpendicular unit vector to it, and $\xi_\|$ and $\xi_\perp$ are the drag coefficients in the parallel and perpendicular directions. Using the facts that (i) $\hat{t} = \frac{\partial \hat{r}}{\partial s}$, where $\hat{r}$ is a unit vector in the direction of the radius vector and the differentiation is along the curve by the reduced curve length parameter s; $s \in \{0, 1\}$, and (ii) that $\hat{n}\hat{n}^T = \boldsymbol{I} - \hat{t}\hat{t}^T$ one gets:

$$\overrightarrow{F}_{hydro} = \xi_\perp \overrightarrow{v} + (\xi_\| - \xi_\perp)(\tfrac{\partial \hat{r}^T}{\partial s} \cdot \overrightarrow{v})\tfrac{\partial \hat{r}}{\partial s} \tag{4}$$

Next, using the facts that (i) for a bent rod without torsion $\frac{\partial \overrightarrow{M}}{\partial l} = EI\frac{\partial \overrightarrow{r}}{\partial l} \times \frac{\partial^3 \overrightarrow{r}}{\partial l^3}$, and (ii) that $s = \frac{1}{L}l$, then, by inserting eq. 4 to eq. 1 one obtains:

$$\frac{\partial \overrightarrow{F}_{elastic}}{\partial s} = (-\xi_\perp \overrightarrow{v} - (\xi_\| - \xi_\perp)(\frac{\partial \hat{r}^T}{\partial s} \cdot \overrightarrow{v})\frac{\partial \hat{r}}{\partial s}) \tag{5}$$

and

$$\frac{EI}{L^2}\frac{\partial^2 \theta}{\partial s^2}\hat{z} = \overrightarrow{F}_{elastic} \times \frac{\partial \hat{r}}{\partial s} \tag{6}$$

To solve these equations we define the dimensionless variables (i) $\overrightarrow{\mathtt{F}} \equiv \frac{L^2}{EI}\overrightarrow{F}_{elastic}$ and (ii) $\sigma \equiv \frac{(\xi_\parallel - \xi_\perp)}{\xi_\perp}$. We also assume without a loss of generality that $\overrightarrow{v} = v_0\overrightarrow{\eta}$ where $\overrightarrow{\eta}$ is a dimensionless function, and define the additional dimensionless variable $\mathtt{V} \equiv \frac{\xi_\perp L^2}{EI}v_0$. Using these relations we obtain:

$$\frac{\partial\overrightarrow{\mathtt{F}}}{\partial s} = -\mathtt{V}\overrightarrow{\eta} - \sigma\mathtt{V}(\frac{\partial \hat{r}^T}{\partial s}\cdot\overrightarrow{\eta})\frac{\partial\hat{r}}{\partial s} \quad (7)$$

and

$$\frac{\partial^2\theta}{\partial s^2}\hat{z} = \overrightarrow{\mathtt{F}} \times \frac{\partial\hat{r}}{\partial s} \quad (8)$$

For a close duct like the microfluidic channel that was used in the experiment, the velocity field is equal to: $\overrightarrow{\eta} = -\eta(y(s), z)\hat{x}$ (see figure 1 of the main text). Using this relation, three coupled differential equations can be derived:

$$\frac{\partial^2\theta}{\partial s^2} = \mathtt{F_x}\cos(\theta) + \mathtt{F_y}\sin(\theta) \quad (9)$$

$$\frac{\partial\mathtt{F_x}}{\partial s} = \mathtt{V}\eta(y(s), z) + \sigma\mathtt{V}\sin^2(\theta)\eta(y(s), z) = (1 + \sigma\sin^2(\theta))\mathtt{V}\eta(y(s), z) \quad (10)$$

$$\frac{\partial\mathtt{F_y}}{\partial s} = -\sigma\mathtt{V}\cos(\theta)\sin(\theta)\eta(y(s), z) \quad (11)$$

In particular for $e/h \ll 1$ the flow profile in a duct is[3]:

$$\eta = \left[1 - (2Z-1)^2 + \sum_{p=1}^{\infty}(-1)^p\frac{32}{(2p-1)^3\pi^3}\frac{\cosh((2p-1)\pi(h/e)(Y-\frac{1}{2}))}{\cosh((2p-1)\pi/2(h/e))}\cos((2p-1)\pi(Z-\frac{1}{2}))\right] \quad (12)$$

where $Z \equiv z/e$ and $Y \equiv y/h$; h being the width of the duct and e its height, $\hat{z}$ the axis along the duct short cross-section dimension and $\hat{y}$ the axis along the duct long cross-section dimension (see figure 1 of the main text) .

Solution of the equations using the Matlab function bvp4c

We solved equations 9-11 with the Matlab function bvp4c using the flexural rigidity as a free variable.
First, a simpler problem was solved where the flow profile inside the duct was assumed to be flat. That is: $\eta(y, z) = 1$. We also define $\overrightarrow{\mathtt{F}^\star} \equiv \frac{L^2}{(EI)_\star}\overrightarrow{F}_{elastic}$ and $\mathtt{V}^\star \equiv \frac{\xi_\perp L^2}{(EI)_\star}v_0$ where $(EI)_\star = 2.8 \times 10^{-20}\ Nm^{-2}$, the value of the flexural rigidity that was measured in ref. [4] . In that case, equations 9-11 are reduced to:

$$\frac{\partial^2\theta}{\partial s^2} = \lambda(\mathtt{F}^\star_\mathtt{x}\cos(\theta) + \mathtt{F}^\star_\mathtt{y}\sin(\theta)) \quad (13)$$

$$\frac{\partial \mathtt{F}^{\star}_{\mathtt{x}}}{\partial s} = (1 + \sigma \sin^2(\theta))\mathtt{V}^{\star} \tag{14}$$

$$\frac{\partial \mathtt{F}^{\star}_{\mathtt{y}}}{\partial s} = -\sigma \mathtt{V}^{\star} \cos(\theta) \sin(\theta) \tag{15}$$

where σ was calculated from reference [5], $\mathtt{V}^{\star}$ was calculated from the infusion rate, and $EI \equiv (EI)_{\star}/\lambda$, with λ a unitless factor.

As mentioned in the main text, a note should be made regarding the boundary conditions [1]. At the free end of the cell the elastic forces and the moments of the forces on it are zero, and hence $\mathtt{F}^{\star}_{\mathtt{x}}(1) = \mathtt{F}^{\star}_{\mathtt{y}}(1) = \frac{\partial \theta(1)}{\partial s} = 0$. On the exist point of the cell from the growth channel, the boundary conditions should be chosen carefully. Analysis of the profiles of cells at that end show that the angle at that point can be different from zero. In addition, the x position of the midcell at the exist from the growth channel can differ from the middle of the growth channel. Hence, cells are not clamped or hinged at that point, but rather are supported (or at least partly supported). In, fact, when a force that was too large was applied on cells, they slipped out of the channel, a fact that supports the conclusion that they are supported at that point. However, for a supported rod, there is no way to a-priori set the boundary conditions at this point [1]. Hence, for each cell, the direction of the cell midline at the exist from the growth channel was chosen according to the actual angle that it had at that point (θ_0). Finally, another boundary conditions was chosen to take into account the free parameter in the flexural rigidity (λ), This condition was that $\theta(1)$ should be equal to the actual angle that the cell had at that point (θ_{max}). Collecting all this information together, we obtain the following boundary conditions:

$$\begin{aligned} \theta(0) &= \theta_0 \\ \theta(1) &= \theta_{max} \\ \tfrac{\partial \theta}{\partial s}(1) &= 0 \\ \mathtt{F}^{\star}_{\mathtt{x}}(1) &= 0 \\ \mathtt{F}^{\star}_{\mathtt{y}}(1) &= 0 \end{aligned} \tag{16}$$

To actually solve this boundary problem, the encoding $\theta \equiv q_1$, $\frac{\partial \theta}{\partial s} \equiv q_2$, $\mathtt{F}^{\star}_{\mathtt{x}} \equiv q_3$, $\mathtt{F}^{\star}_{\mathtt{y}} \equiv q_4$ was used. The initial guess for the shape of the cells were chosen as a sigmoid, the initial guess for the force in the $\hat{x}$ direction was chosen to be a linearly increasing function and that of the force in the $\hat{y}$ direction to be a parabolically increasing one.

After solving the above simplified problem. The full problem was solved with $\eta(y, z)$ taking from eq. 12, using the results for $\theta(s)$, $\mathtt{F}^{\star}(s)$ and λ of the simplified problems as the initial guesses for their values. There are however three points that should be considered that results from the fact that the force at each point on the cell is different. First, as was mentioned in the text, cells tend to grow out of the focal plane. and a z stack was used in order to calculate the location of the midlife of the cell. Since most of the cells reside in a height of $0 - 4$ μm ($0 \leq Z \leq 4/26$) above the bottom of the main channel, we integrated out the dependence on this variable from eq. 12 and obtain:

$$\eta = \left[\eta_0 + \sum_{p=1}^{8} H(p)\cos((2p-1)\pi(Y-\frac{1}{2}))\right] \tag{17}$$

where:

$$\eta_0 \equiv \frac{e}{e_0}\int_0^{e_0/e} \{1-(2Z-1)^2\}dZ \tag{18}$$

and

$$H(p) \equiv \frac{e}{e_0}\int_0^{e_0/e} (-1)^p \frac{32}{(2p-1)^3\pi^3}\frac{\cos((2p-1)\pi(Z-\frac{1}{2}))}{\cosh((2p-1)\frac{\pi}{2}(h/e))}dZ \tag{19}$$

here e_0 is the upper point that the cell may reach that was taken to be 4 μm above the channel surface and the sum was truncated after eight terms to assure finite calculation time.

As for the second point, recall that $v = v_0\eta$. Hence, in order to solve this problem and still make sure that the value of the velocity is calculated in the right way, we used in this case a value of v_0 so that $v_0 \times \int\int \eta dZdY = v_0^{Flat}$ where v_0^{Flat} is the velocity for a flat flow profile as is calculated from the infusion rate.

The third point arises from the fact that there is no simple way to let bvp4c to use values on the LHS of eq. 9 -11 that depend on the point in the $\hat{y}$ direction where the current step of bvp4c is calculated. In order to solve this problem we used the fact that the y coordinate of the point that bvp4c uses for the current step can be calculated for the relation $y = \int L\cos(\theta(s))ds$, and hence $dy = L\cos(\theta)ds$. Hence, an additional dummy equation can be used

$$\frac{dY}{ds} = \cos(\theta)\mathcal{L} \tag{20}$$

where $\mathcal{L} \equiv L\backslash e$.

Thus, by defining $q_5 \equiv Y$, we have used bvp4c to solve the following equations:

$$\begin{array}{c} \frac{\partial q_1}{\partial s} = q_2 \\ \frac{\partial q_2}{\partial s} = \lambda\left(q_3\cos(q_1) + q_4\sin(q_1)\right) \\ \frac{\partial q_3}{\partial s} = \mathtt{V}^\star(1+\sigma\sin^2(q_1))\left[\eta_0 + \sum_{p=1}^{8} H(p)\cosh((2p-1)\pi(h/e)(q_5-\frac{1}{2}))\right] \\ \frac{\partial q_4}{\partial s} = -\sigma\mathtt{V}^\star\cos(q_1)\sin(q_1)\left[\eta_0 + \sum_{p=1}^{8} H(p)\cosh((2p-1)\pi(h/e)(q_5-\frac{1}{2}))\right] \\ \frac{dq_5}{ds} = \cos(\theta)\mathcal{L} \end{array} \tag{21}$$

with the boundary conditions:

$$
\begin{aligned}
q_1(0) &= \theta_0 \\
q_1(1) &= \theta_{max} \\
q_2(1) &= 0 \\
q_3(1) &= 0 \\
q_4(1) &= 0 \\
q_5(0) &= 0
\end{aligned}
\tag{22}
$$

and the flexural rigidity was calculated from the output of bvp4c function.

Finally, in order to obtain the error in the estimation of the flexural rigidity, eq. 21 was solved again twice, with the initial conditions:

$$
\begin{aligned}
q_1(0) &= \theta_0 - \Delta\theta_0 & & & q_1(0) &= \theta_0 + \Delta\theta_0 \\
q_1(1) &= \theta_{max} - \Delta\theta_{max} & & & q_1(1) &= \theta_{max} + \Delta\theta_{max} \\
q_2(1) &= 0 & & or & q_2(1) &= 0 \\
q_3(1) &= 0 & & & q_3(1) &= 0 \\
q_4(1) &= 0 & & & q_4(1) &= 0 \\
q_5(0) &= 0 & & & q_5(0) &= 0
\end{aligned}
\tag{23}
$$

where $\Delta\theta_0$ and $\Delta\theta_{max}$ are the errors of the actual angles of the cell at its beginning and end as obtain from the fits to its beginning and end sections. The error of the flexural rigidity was estimated as the standard deviation of the flexural rigidities as obtain from bvp4c in the three cases.

References

[1] L.D. Landau & E.M. Lifshitz, Theory of Elasticity (Volume 7 of A Course of Theoretical Physics), Forth edition, Pergamon Press 1970.

[2] Henry C. Fu, Chapter 1 - Fluid–Structure Interactions and Flagellar Actuation, In: Minjun Kim, Edward Steager and Agung Julius, Editor(s), Microbiorobotics, William Andrew Publishing, 2012, Pages 3-28.

[3] Gondret, P., N. Rakotomalala, M. Rabaud, D. Salin, P. Watzky. 1997. Viscous parallel flows in finite aspect ratio Hele-Shaw cell: Analytical and numerical results. Phys. Fluids 9:1841–1843

[4] Wang, S., Arellano-Santoyo, H., Combs, P.A., and J. W. Shaevitz. 2010. Actin-like cytoskeleton filaments contribute to cell mechanics in bacteria. PNAS. 107: 9182–9185

[5] Tirado, M. M., C. L. Martfnez, and J. G. de la Torre. 1984. Comparison of theories for the translational and rotational diffusion coefficients of rod-like macromolecules. Application to short DNA fragments. J. Chem. Phys. 81:2047–2052
